# Enhancing Pigment Phenotyping and Classification in Lettuce through the Integration of Reflectance Spectroscopy and AI Algorithms

**DOI:** 10.3390/plants12061333

**Published:** 2023-03-16

**Authors:** Renan Falcioni, João Vitor Ferreira Gonçalves, Karym Mayara de Oliveira, Caio Almeida de Oliveira, José A. M. Demattê, Werner Camargos Antunes, Marcos Rafael Nanni

**Affiliations:** 1 Graduate Program in Agronomy, Department of Agronomy, State University of Maringá, Av. Colombo, 5790, Maringá 87020-900, Paraná, Brazil; pg403805@uem.br (J.V.F.G.); pg54640@uem.br (K.M.d.O.); pg403699@uem.br (C.A.d.O.); wcantunes@uem.br (W.C.A.); mrnanni@uem.br (M.R.N.); 2 Department of Soil Science, Luiz de Queiroz College of Agriculture, University of São Paulo, Av. Pádua Dias, 11, Piracicaba 13418-260, São Paulo, Brazil; jamdemat@usp.br

**Keywords:** artificial intelligence, crop species, hyperspectral technology, lettuce plant varieties, pigment phenotyping in agriculture, sustainable practices, wavelengths

## Abstract

In this study, we investigated the use of artificial intelligence algorithms (AIAs) in combination with VIS-NIR-SWIR hyperspectroscopy for the classification of eleven lettuce plant varieties. For this purpose, a spectroradiometer was utilized to collect hyperspectral data in the VIS-NIR-SWIR range, and 17 AIAs were applied to classify lettuce plants. The results showed that the highest accuracy and precision were achieved using the full hyperspectral curves or the specific spectral ranges of 400–700 nm, 700–1300 nm, and 1300–2400 nm. Four models, AdB, CN2, G-Boo, and NN, demonstrated exceptional R^2^ and ROC values, exceeding 0.99, when compared between all models and confirming the hypothesis and highlighting the potential of AIAs and hyperspectral fingerprints for efficient, precise classification and pigment phenotyping in agriculture. The findings of this study have important implications for the development of efficient methods for phenotyping and classification in agriculture and the potential of AIAs in combination with hyperspectral technology. To advance our understanding of the capabilities of hyperspectroscopy and AIs in precision agriculture and contribute to the development of more effective and sustainable agriculture practices, further research is needed to explore the full potential of these technologies in different crop species and environments.

## 1. Introduction

*Lactuca sativa* L., also known as lettuce, is a nutritionally important food for the population [1,2]. It is a popular and economically significant vegetable consumed worldwide, with an estimated production of 27 million tons according to the Food and Agriculture Organization (FAO) in 2022 [3]. The classification and production estimation of green, green-purplish, and purple lettuce varieties have been the focus of several studies to increase production [3,4,5].

In Brazil, various lettuce varieties, genotypes, and phenotypes exhibit great potential for the automation of classification due to the presence of green to purple pigments, antioxidant pigments, and sensory characteristics [6,7]. Furthermore, the leaves of these plants exhibit a range of colours, including green, orange, violet, and purple, which are associated with one or more biomolecules and antioxidant compounds, affecting the classification of these plants [3,4]. Thus, the rapid, precise, and accurate pigment phenotyping of lettuce varieties is of significant interest for both vertical and indoor farming and traditional agriculture and greenhouse production [4,5,6,7,8].

In recent years, horticultural and postharvest crops have been studied for the classification of phenotypes, varieties, and genetic crop plant analysis [9,10]. In this way, the use of remote sensing tools such as RGB tools, Multispectral Image Sensors (MSI), Hyperspectral Imaging Sensors (HSI), and Visible–Near-Infrared–Shortwave-Infrared (VIS-NIR-SWIR) spectroscopy combined with artificial intelligence algorithms (AIAs) can improve the accuracy and precision of crop classification [10,11]. This is of great importance, considering that the global food economy is estimated at approximately $750 billion annually, and AIAs can help reduce food waste and improve consumer satisfaction. Accordingly, the integration of AIAs and hyperspectral data mining, deep learning, and machine learning techniques promises to be a promising approach for crop plant classification [1,7,12].

The application of remote sensing 5.0 techniques in crop science is crucial for the phenotyping and quantification of pigments using leaf-based spectroscopy [13,14]. For example, the speed, precision, and accuracy of spectroradiometer equipment, combined with the capacity of multivariate and artificial intelligence algorithms (AIAs) for modelling, results in improved analysis of crop classification [8,11,14]. This allows for advances in accuracy in monitoring crop development and growth [13], as well as other structural and water-related characteristics of leaves [9,13,15]. In this sense, the integration of AI algorithms for rapid classification and learning, encompassing morphological, genetic, biochemical, and physiological attributes, presents a new perspective in remote sensing in crop science [1,16,17]. Accordingly, the highest accuracy of the model can then be used to classify agronomically relevant varieties in large sample sets based on specific spectral signatures and AIAs linked to deconvolution wavelengths [3,18,19,20].

Deep learning and machine learning are artificial intelligence techniques that have significant applications in agriculture, including the analysis and classification of agronomic crops [4,7]. These techniques enable computers to learn from data and make predictions or decisions without being explicitly programmed [4,6]. In crops such as wheat, maize, and soybean, they can be used to analyse large amounts of data from sensors, drones, or satellite images, to detect patterns or anomalies, and to optimize irrigation, fertilization, or pest control [15,21,22,23]. In lettuce plants, they can be used to analyse hyperspectral data or ATR-FTIR spectroscopy data to accurately classify lettuce varieties, and to predict crop yield or quality [15,23]. Deep learning is a specific type of machine learning that can be particularly useful for these tasks, as it uses artificial neural networks to process and analyse data, and can learn to recognize complex patterns in the environment [24,25,26].

**Figure 1:**
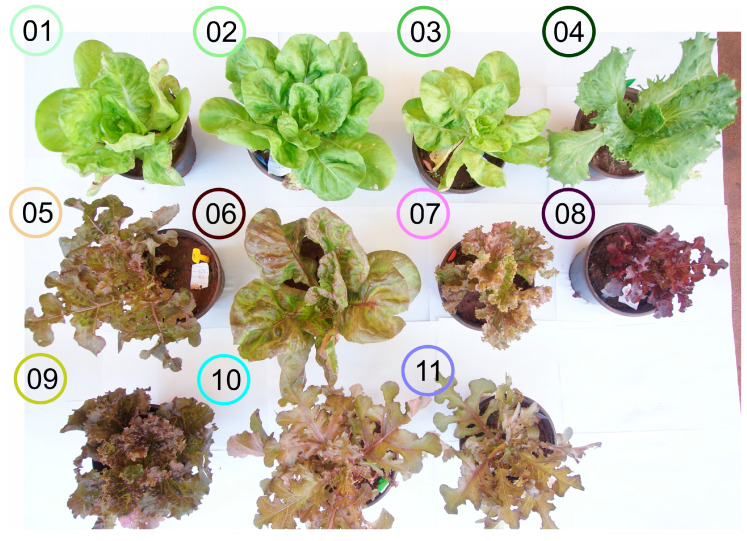
*Lactuca sativa* L. plants with variation of pigments. The circle displays eleven different varieties of the **left** to **right** and **top** to **bottom** (**V01**–**V11**) variations as described in the Section 4 [3,8].

The objective of this study was to assess the efficacy of 17 high-throughput classifications combined with artificial intelligence algorithms (AIAs) and machine learning for lettuce plants using hyperspectroscopy in the VIS-NIR-SWIR range. The hypothesis of this study was to determine the wavelengths most significant for pigment phenotyping and to classify 11 varieties of lettuce plants (Figure 1) using artificial intelligence algorithms (AIAs). For this purpose, spectroradiometers and AIAs were trained and tested on green, green-purplish, and purple lettuce plants using remote sensing tools in the VIS (400–700 nm), NIR (700–1300 nm), SWIR (1300–2400 nm), and full spectra (400–2400 nm) ranges.

## 2. Results

### 2.1. Variance and Descriptive Analysis-Based Biochemical Attributes of Lettuce

**Figure 2:**
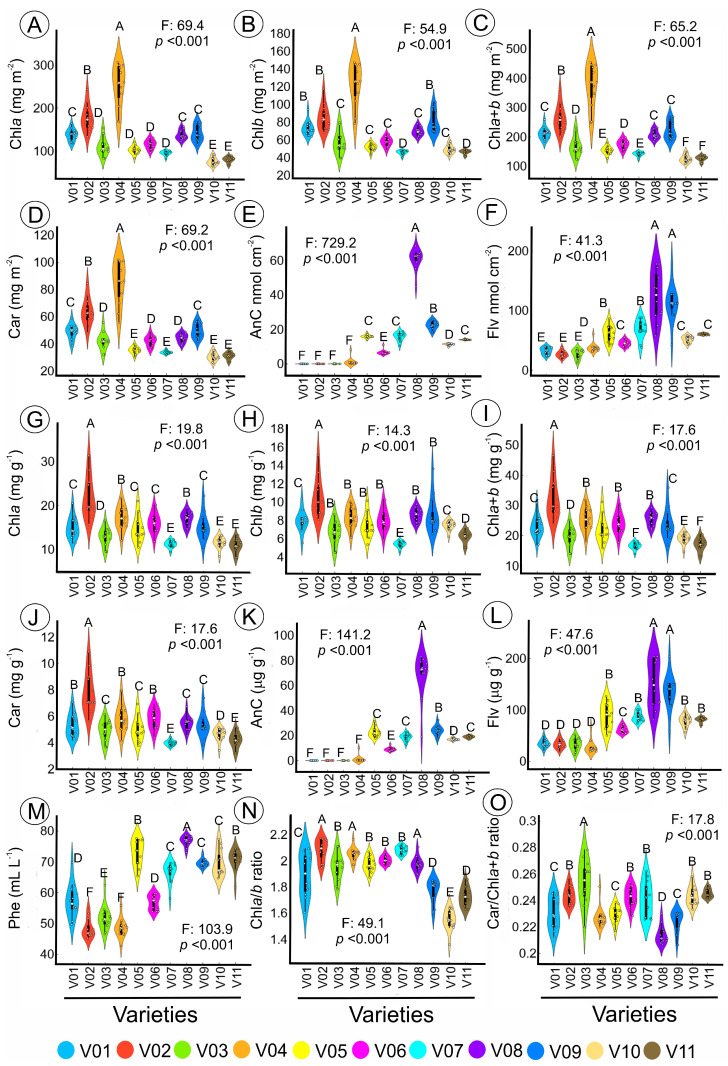
Violin plot of leaf pigment concentration expressed by (**A**–**F**) leaf area (mg m^−2^), (**G**–**L**) mass (mg g^−1^), (**M**) volume (mL L^−1^) and (**N**,**O**) ratio units. Different letters over the violin plot indicate significant differences by Duncan’s test (*p* < 0.001) between lettuce plants. Abbreviation in Section 4 (*n* = 132).

The analysis of the genetic diversity of phenotypes in green, green-purplish, and purple leaves is presented in Figure 2. The variations in pigment concentration were evaluated in terms of base area, mass, and volume and showed significant differences (*p* < 0.001). The violin plot indicates that the chlorophyll (Chl) and carotenoid (Car) concentrations expressed in the base area were higher in lettuce varieties V01–V04 and lower in varieties V07, V10, and V11 (*p* < 0.001). The purple lettuce varieties (V08 and V09) consistently showed higher increases in anthocyanin (AnC) and flavonoids (Flv) in the base area and mass compared to green-purplish lettuce plants. Green-purplish varieties had low levels of AnC, Flv, and phenol (Phe), while green varieties had extremely low levels (Figure 2E,K). All 15 parameters analysed were found to be significantly different (F-test: 17.6 at 729.2; *p* < 0.001), as shown in Figure 2A–O.

Table S1 and Figure 2 also display the coefficient of variation (CV%) of the leaf pigment base mass, area, and volume parameters for 11 lettuce varieties. The CV values ranged from 6.1 to 125.8% and the 15 parameters were classified as low to very high. Two parameters were classified as low (Chl*a*/*b* ratio, Car/Chl*a* + *b* ratio), one parameter was medium (Phe(vol)), four parameters were high (Chl*a*;_(mass)_, Chl*b*_(mass)_, Chl*a* + *b*_(mass)_, Car(mass)), and eight parameters were very high (Chl*a*_(area)_, Chl*b*_(area)_, Chl*a* + *b*_(area)_, Car_(area)_, AnC_(area)_, Flv_(area)_, AnC_(mass)_, Flv_(mass)_), as reported in Table S1.

### 2.2. Reflectance Hyperspectral Analysis in Leaves

**Figure 3:**
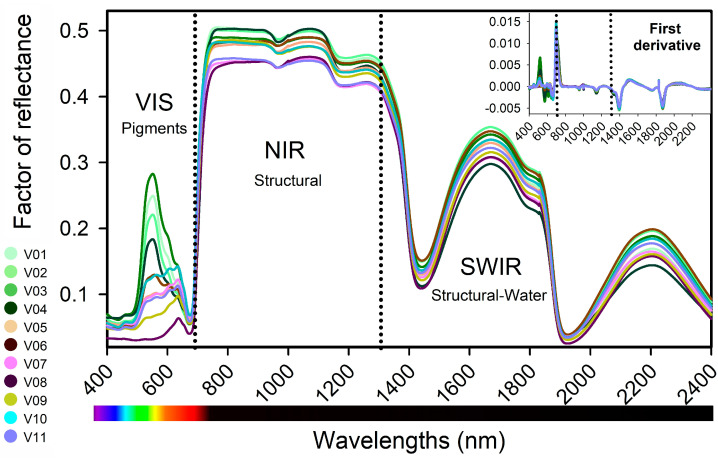
Average foliar VIS-NIR-SWIR reflectance hyperspectral factor of reflectance profiles (400–2400 nm) of V01–V11, lettuce varieties. The inset displays the first derivative of the hyperspectral data. Note delimitation of the inflexion points of 700 and 1300 nm. (*n* = 66). Standard error was omitted for clarity.

Figure 3 displays the VIS-NIR-SWIR hyperspectral data of ≈360 lettuce leaf curves (≈5 curves per biological sample, in total 66 samples). Permutation multivariate analysis of variance indicated significant wavelengths (F: 19.2; *p* < 0.001) in all the spectral data. A range of larger to slight and significant variations in reflectance factor were observed in the VIS region, which are associated with phenolic compounds, flavonoids, anthocyanins, carotenoids, and chlorophyll concentrations. The near-infrared (NIR) region showed differences in the biophysical and biochemical properties of leaf tissues, while the shortwave infrared (SWIR) region was associated with the structural water contents. Transforming the data to the first derivative reduced the scale; however, this transformation, as well as PCA and other analysis methods, showed the lowest performance (Figure 3; inset).

**Figure 4:**
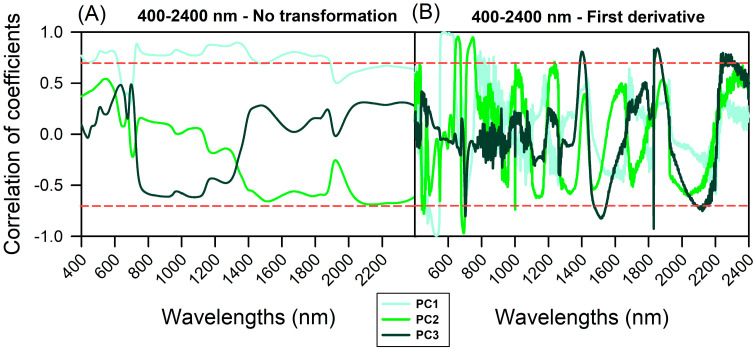
Correlation of coefficients associated with PC1–PC3 (light to dark green lines). (**A**) 400–2400 nm data not transformed. (**B**) 400–2400 nm to first derivative. The red line indicates −0.70 and +0.70 correlation of coefficients.

The relationships between wavelengths and selected principal component (PC) wavelengths in lettuce varieties are presented in Figure 4. There was a significant correlation (*p* < 0.001) between the principal components (PC1–PC3) and the varieties’ differentiation characteristics, both for the raw data (Figure 4A) and first derivative data (Figure 4B).

Phenotyping of lettuce varieties can be accomplished by combining VIS-NIR-SWIR hyperspectroscopy with artificial intelligence algorithms (Figure 4). The strong correlation between the full spectrum or selected wavelength ranges (400–700 nm, 700–1300 nm, and 1300–2400 nm) and vibration hyperspectroscopy suggests the usefulness of hyperspectroscopy for pigment phenotyping (Figure 4). However, the application of artificial intelligence algorithms did not yield a desirable outcome when used on the first derivative data (data not shown).

### 2.3. Cluster Heatmap of Selected Wavelengths and Classification-Based Resolution Bands

**Figure 5:**
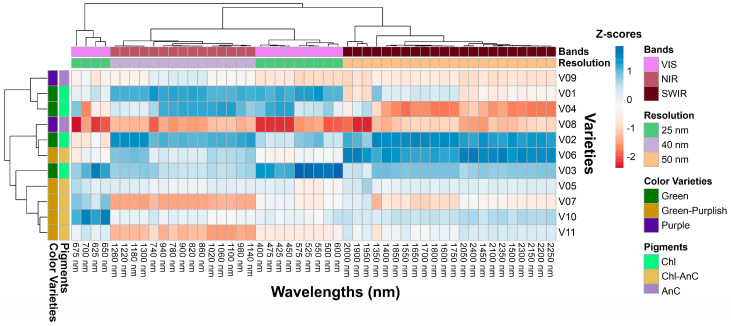
Cluster heatmap shows the correlation between the spectral bands, lettuce varieties, and pigment concentrations (chlorophylls, chlorophylls–anthocyanins, anthocyanins). The correlations are organized by wavelength range (VIS, NIR, SWIR), spectral resolution (25, 40, 50 nm), and colour variety (green, green-purple, purple). Positive relationships are shown in blue (Z-scores *p* < 0.001) while negative correlations are shown in red.

The relationship between the pigments in lettuce plants and the wavelengths of hyperspectral data was analysed using cluster analysis, as illustrated in Figure 5. The colour on the cluster heatmap indicates the correlation between the hyperspectral values and the concentration of pigments, with green representing chlorophylls, green-purple representing the combination of chlorophyll and anthocyanin, and purple representing anthocyanins. Z-scores were used to reflect the variability of the data, with blue colour showing a correlation slope of approximately 2, red colour indicating a slope of approximately −2, and light colour indicating a weak association of approximately 0. A deeper shade of blue for a particular wavelength band indicated that 11 varieties had a higher concentration of a specific pigment and higher reflectance signals at that wavelength band compared to varieties with low pigment expression (Figure 5).

A deeper shade of red indicated that varieties with higher anthocyanin, flavonoid, and phenolic concentrations had higher reflectance signals at the wavelength bands V08, V10, and V11 compared to varieties with lower pigment concentrations (Figure 5). Light shades indicated that some wavelengths were not effective in profiling the foliar content of specific pigments (V05 and V10). Despite their high presence in the leaves, the pigments Car, AnC, Flv, and Phe displayed a distinct correlation pattern in the NIR-SWIR range, which was linked to the structure and water content of the leaves.

### 2.4. Principal Component Analysis (PCA)

**Figure 6:**
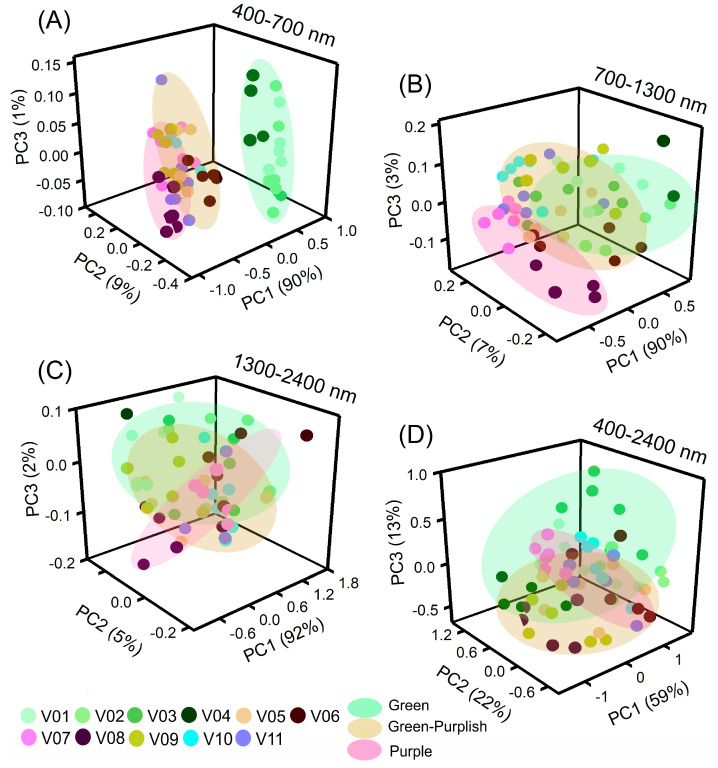
(**A**) Principal component (PC) analysis by 3D-plot for PC1, PC2, and PC3 of the hyperspectroscopy data from lettuce plant varieties. (**A**) 400 at 700 nm. (**B**) 700 at 1300 nm. (**C**) 1300 at 2400 nm. (**D**) 400 at 2400 nm. Green, orange, and pink colour display clusters of lettuce varieties (*n* = 66).

The clustering analysis of full-spectral bands for lettuce varieties is presented in Figure 6. The PCA-AIA dataset based on artificial intelligence algorithms for the spectral ranges 400–700 nm, 700–1300 nm, 1300–2400 nm, and 400–2400 nm (represented as PC1, PC2, PC3, and PC4, respectively) explained 100%, 100%, 99%, and 94% of the total variance, respectively.

### 2.5. Artificial Intelligence Algorithm (AIA)-Based Data Mining and Deep Machine Learning for Classified Lettuce

**Figure 7:**
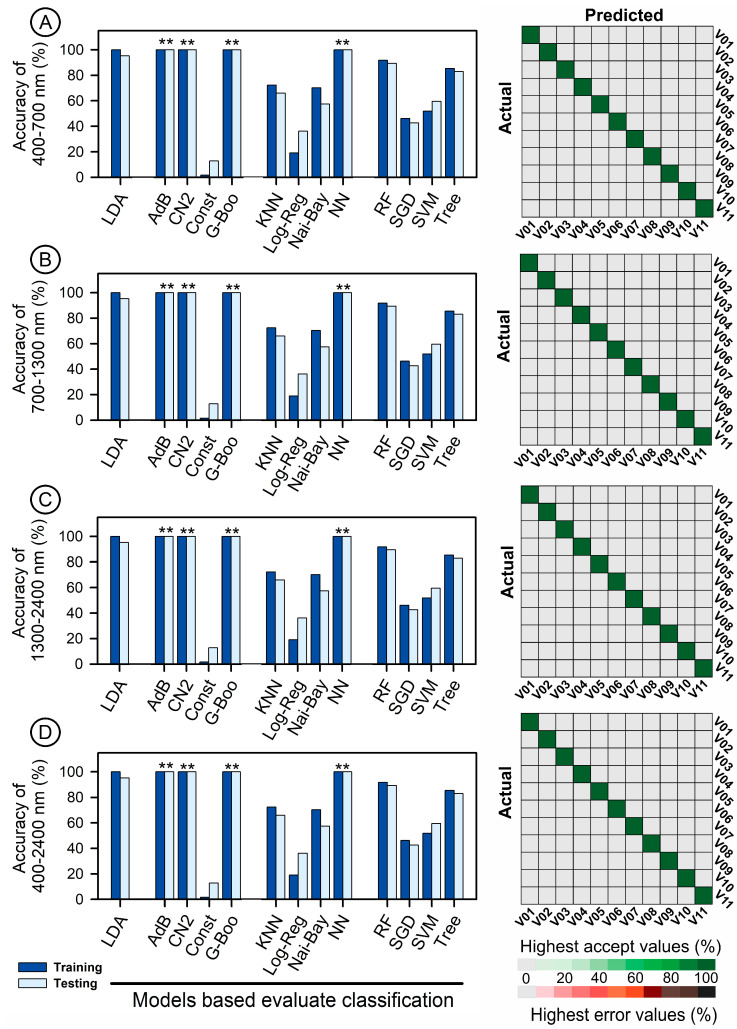
Evaluation of accuracy in training and testing for 17 algorithms including LDA, AdB, CN2, Const, G-Boo, KNN, Log-Reg, Nai-Bay, NN, RF, SGD, SVM, and Tree using VIS-NIR-SWIR hyperspectral data for lettuce phenotypic characterization. (**A**) Spectral range of 400–700 nm. (**B**) Spectral range of 700–1300 nm. (**C**) Spectral range of 1300–2400 nm. (**D**) Spectral range of 400–2400 nm. ** asterisk shows accuracy below 99.9% and accepts values for classification based on those algorithms (*n* = 66).

Figure 7 presents the evaluation of phenotypic characteristics through VIS-NIR-SWIR hyperspectral data. Seventeen algorithms, including linear discriminant analysis (LDA), adaBoost (Adb), CN2 rule inducer (CN2), constant (Const), gradient boosting (G-Boo), kernel k nearest neighbours (KNN), logistic regression (Log-Reg), naive Bayes (Nai-Bay), neural network (NN), random forest (RF), stochastic gradient descent (SGD), support vector machine (SVM), and tree (Tree), were used to classify lettuce plants and showed performance ranging from low to high.

The algorithms were applied to VIS-NIR-SWIR spectra to classify the training and testing models using cross-validation data (Figure 7A–D). The AdB, G-Boo, CN2, and NN algorithms achieved 100% accuracy with high precision and required a relatively short evaluation time. In addition, the algorithms and confusion matrix displayed medium accuracy and precision for LDA, RF, Tree, and Nai-Bay, with accuracy and precision ranging from 64% to 89% for the training and testing models.

On the other hand, SGD, SVM, and Const showed lower accuracy, with less than 45% accuracy for the training and 51% for the testing models. This suggests that either full range bands or individual wavelength values hold potential for precise classification using computational intelligence techniques (Figure 7).

The results of hyperspectral analysis using VIS-NIR-SWIR spectral bands and the STEPDISC methodology showed that the 11 varieties of lettuce were successfully classified using artificial intelligence techniques. The selection of 139 wavelengths was based on F-value, partial R^2^, and average squared canonical correlation criteria. For example, some models were created through the calibration of the sample spectral curves and were defined by contingency coefficients (R^2^ = 0.967) using the STEPDISC algorithm. In this way, the validity of the models was evaluated using independent samples in the discriminant models and was found to be highly significant (*p* < 0.001). In this sense, the results showed high correlations in the correctly classified models, with 65.5% accuracy (R^2^ = 0.843) for VIS, 82.2% accuracy (R^2^ = 0.961) for NIR, 66.9% accuracy (R^2^ = 0.668) for SWIR, and 78.5% accuracy (R^2^ = 0.813) for VIS-NIR-SWIR, as reported in Table 1.

### 2.6. STEPWise (STEPW) and Variable Importance to the Projection (VIP) to Selection Wavelengths

The performance of artificial intelligence algorithms was evaluated based on the STEPW and VIP values for 22 to 57 wavelengths. These results indicated that the STEPW effectively discriminated multiple wavelengths in the visible region, including blue (445 nm), green (555 nm), and red (660 nm) in the near-infrared (NIR) region and red edge (699–750 nm), NIR (940 nm, 1040 nm, and 1330 nm), SWIR (1800 and 2210 nm), as shown in Table S2. Meanwhile, VIP values discriminated visible wavelengths such as green (555 nm), red (610 nm), red edge (710 nm and 750 nm) in the NIR region, NIR (890 nm, 960 nm, and 1150 nm), and SWIR (1410, 1830, 2340 nm), as outlined in Table S2.

Algorithms including PCA, HC, LDA, AdB, CN2, G-Boo, Nai-Bay, NN, RF, and Tree demonstrated excellent classification performance when used with STEPW and VIP values. However, algorithms such as Const, KNN, Log-Reg, SGD, and SVM showed lower performance in terms of correct classification, resulting in up to 80% loss in precision and accuracy during training and testing models for lettuce plants (as demonstrated in Tables S2 and S3 and reported in Figure 7).

## 3. Discussion

### 3.1. Descriptive and Variance Analyses of Lettuce Varieties

Efficient classification and estimation of lettuce plants in green, green-purplish, and purple colour was achieved using 17 artificial intelligence algorithms in the VIS-NIR-SWIR bands, as shown in Table 1 and Table S1–S3 and Figure 1, Figure 2, Figure 3, Figure 4, Figure 5, Figure 6 and Figure 7. The accuracy of some algorithms reached over 90% after being trained and tested, as seen in Figure 7. This confirms the significance of using machine learning (ML), deep learning (DL), and data mining (DM) in AI tools for high-throughput pigment phenotyping screening of 11 lettuce varieties, as previously reported [1,18,27,28]. To enhance accuracy, it is recommended to combine higher concentrations of Chl, Car, AnC, Flv, and Phe in leaves with leaf thickness. In this sense, research, based on differences in biochemical and biophysical parameters, particularly in NIR-SWIR spectra, suggests that AI tools can effectively discriminate between lettuce varieties [1,18,29].

Indoor farming of lettuce using hyperspectroscopy has been shown to be effective in monitoring and diagnosing the growth and development of the plants [1,3]. Research supports the use of artificial intelligence (AI) tools in combination with VIS-NIR-SWIR spectroscopy for accurately classifying lettuce varieties in vertical farms, hydroponic systems, and traditional agriculture, as demonstrated by the results presented in Table 1 and Table S1–S3 and Figure 1, Figure 2, Figure 3, Figure 4, Figure 5, Figure 6 and Figure 7 [3,19,30].

### 3.2. Analysis of the Hyperspectral and Fingerprint Curves

The application of visible (VIS), near infrared (NIR), and shortwave infrared (SWIR) hyperspectroscopy has been proposed as an efficient and non-destructive method for classifying and measuring the biochemical and compound properties of plant leaves. Accordingly, VIS bands highlight variations in pigment absorbance, including chloroplastidic pigments (Cars and Chls) and extrachloroplastidic pigments (vacuolar or cytoplasmatic pigments such as AnCs, Flvs, and Phes) [29].

The 700–1300 nm range has been found to have higher reflectance values and is believed to reflect differences in the molecular, biochemical, anatomical, and physiological characteristics of plants [1,3]. For instance, previous studies have correlated variations in the thickness of parenchyma cells in different lettuce varieties to differences in radiation scattering [31,32]. On the other hand, SWIR bands, composed mainly of organic compounds such as structural carbohydrates, amino acids, and proteins also demonstrate major variations in vibrational spectra [20].

In food and crop science, accurately measuring chemical compositions is essential for ensuring production and consumer safety as well as for pigment phenotyping through computational algorithms. In this way, principal component analysis (PCA) clusters have been shown to reflect hyperspectral data and variations in pigment structure components and structure–water bands. Therefore, by utilizing multiple bands and AI algorithms, misclassification problems can be resolved, leading to the production of higher quality vegetables, grains, and fruits as well as correct classification [1,3,4,8,33,34].

### 3.3. Artificial Intelligence Algorithms (AIAs) for Rapid and Precise Classification

AI algorithms (AIAs) applied to classify sample groups using hyperspectral data show promising results [3,19,35]. The AdB, CN2, G-Boo, and NN models improved precision with R^2^, precision–recall, and ROC area values greater than >0.99 from reflectance spectroscopy curves.

Despite the expectation that HC, LDA, KNN, Log-Reg, Nai-Bay, RF, SGD, SVM, and Tree [12,31] would have the highest precision and accuracy, our study found otherwise. Instead, SVM, PLS, and RF algorithms showed the highest accuracy for lettuce pigment phenotyping when used with a regression model [20,36]. In this sense, and accordingly [3,15], reflectance hyperspectral methods showed higher accuracy, with LDA-linear carrying 99.9% of the data variance [3].

Our study proposed that simple methods combined with AIAs (AdB, CN2, G-Boo, and NN algorithms) can classify lettuce varieties with high accuracy and precision based on results from at least eleven lettuce plants [1]. Therefore, algorithms such as iPLSR, PLSR, LDA, RF, SGD, and SVR over VIs were used to predict and classify optical leaf properties with high precision [12,29]. Some research reports successful prediction of pigments by VIs, with models developed by the best-performing RF, KNN, SGD, and Tree algorithms.

The significance of AIAs in correct plant classification has increased with the advancement of agriculture 4.0 and 5.0. Although SVR modelling can be performed efficiently, this was not observed in our study (Figure 6). Accordingly, neural network, random forest, and lasso/ridge regression are other AI and learning approaches that could contribute to R^2^, ROC, precision, and accuracy (>0.99) classification of lettuce plants. However, LogLoss showed 1.66 and 0.007 for CN2 and NN, although these algorithms did not show a reduction in the accuracy of the models (Figure 7).

### 3.4. STEPWise (STEPW) and Variable Importance to the Projection (VIP) to Selection Wavelengths

In this study, our objective was to evaluate the efficiency of AI algorithms (AIAs) and machine learning techniques in classifying and pigment phenotyping crops and cultivated species, including native species, corn, tobacco, wheat, cucurbits, lettuce, and others. Our focus was on analysing their biochemical compounds and pigments, with most of the studies being conducted at wavelengths less than 400–700 nm.

Accordingly, the results showed that STEPW and VIP are useful in phenotyping plants, as reported in previous studies [31,33,37]. However, it has been noted that these methods have limitations, such as low accuracy and biases in the output. On average, our findings indicate that 11.5% of the STEPW and VIP values were shared among the range of data, with VIP values ranging from 5.1 to 20.6%, as reported in [1,26,31].

The selection of appropriate AIAs and spectroscopy methods is essential for their application in food safety and nutrition sciences. The analysis of complex leaf data is necessary to obtain the most accurate information using remote sensing techniques for food safety analysis, as reported in [1,31,38]. Therefore, the choice of AIAs and spectroscopy methods is a crucial factor for their use in food safety and nutrition sciences.

## 4. Materials and Methods

### 4.1. Plant Material

*Lactuca sativa* L. plants were grown in a greenhouse at the State University of Maringá in Maringá, Paraná, Brazil, as described in [3]. For the study, 11 lettuce varieties were selected, including Rainha de Maio (V01), Vitória (V02), Maravilha de Inverno (V03), Grandes Lagos Americana (V04), Mimosa Prado (V05), Quatro Estações (V06), Batávia Joaquina (V07), Mimosa Vermelha (V08), Batávia Cacimba (V09), Pipa (V10), and Mimosa Rubi (V11) (Figure 1). Fertilization was applied with N-P-K (10-10-10) as recommended for lettuce plants. The intensity of the light was monitored and reached 1200 µmol m^−2^ s^−1^ irradiance, measured by an LI-190R quantum sensor (Li-Cor Inc., Lincoln, NE, USA) under a natural photoperiod (approx. 12/12; light/dark) at 30 °C (±5 °C) with 45–85% relative humidity. The cultivation was conducted through a completely randomized design with 11 treatments and 6 biological repetitions (Figure 1).

### 4.2. Pigment Analysis and Reflectance Hyperspectral Data

Pigment profiling was performed to extract and quantify chlorophylls, carotenoids, anthocyanins, flavonoids, and phenolic concentrations in methanolic solutions, as described in previous studies [1,3]. Reflectance hyperspectroscopy was measured over the 400–2400 nm range using an ASD FieldSpec 3 spectroradiometer (ASD Inc., Longmont, CO, USA). The light beam was calibrated with a standard Spectralon^®^ for correct measurement as previously described [19,33,39].

### 4.3. Statistical and Graphical Analyses

#### 4.3.1. Statistical Analyses

In the field of scientific research, various statistical and graphical analysis methods are used to better understand and interpret data [33,40]. Descriptive statistics were calculated, and one-way ANOVA for mean comparisons was performed to determine statistical significance (*p* < 0.001) with post hoc Duncan’s test applied to compare biochemical attributes. Pearson’s correlation was also performed using various software programs, such as Excel 2021 (Microsoft Office Inc., Redmond, WA, USA), XLSTAT (Addinsoft, Paris, FRA), Statistical Analysis System software (SAS Institute, Inc., Cary, NC, USA), and Statistica 12.0 (Statsoft Inc., Uppsala, SWE).

#### 4.3.2. Analyses by Spectral Fingerprints and Reflectance Spectroscopy in Leaves

The spectral fingerprints of leaves, together with vibrational modes, were associated with different lettuce varieties using SigmaPlot 12.0 (Systat Inc., San Jose, CA, USA). Principal component analysis (PCA) was performed using The Unscramber x10.4^®^ (Camo Software, Oslo, Norway, NOK). The wavelength discriminant statistics were found using the STEPDISC and STEPWise algorithms available in the Statistical Analysis System software (SAS software version 9.4 (SAS Institute, Inc., Cary, NC, USA)).

#### 4.3.3. Data Mining, Deep Learning, and Machine Learning Algorithm Models

For data mining and machine learning, 17 artificial intelligence algorithms (AIAs) were performed using Orange Data Mining 3.33 (Open-Software). These algorithms include principal component analysis (PCA), hierarchical clustering (HC), linear discriminant analysis (LDA), adaBoost (AdB), CN2 rule inducer (CN2), constant (Const), gradient boosting (G-Boo), kernel k nearest neighbours (KNN), logistic regression (Log-Reg), naive Bayes (Nai-Bay), neural network (NN), stochastic gradient descent (SGD), random forest (RF), support vector machines (SVM), tree (Tree), STEPWise (STEPW), and variable importance in projection (VIP), as described in Table S3 [41]. The algorithms were tested based on a proportion of 60:40 (60% training and 40% testing) of the evaluated data, and the results were analysed based on the rank-performed precision and recall data. In addition, the performance of the AIAs was evaluated using confusion matrix data with the R package R-Core-Team (2021) [42] and graphics plotted using a free online platform for data analysis and visualization (https://www.bioinformatics.com.cn/en, accessed on 22 November 2022).

## 5. Conclusions

In conclusion, our study highlights the potential of using hyperspectral analysis and artificial intelligence (AI) for improved pigment phenotyping and classification in lettuce plants. The results demonstrate that the AdaBoost (AdB), CN2, Gradient Boosting (G-Boo), and Neural Network (NN) algorithms achieved high precision and discrimination rates. The use of deep learning techniques, AI algorithms, and data mining in VIS-NIR-SWIR spectroscopy has significant implications for precision agriculture and can be applied to AI systems for classification. This will advance our understanding of the capabilities of hyperspectral analysis and AI for precision agriculture, and contribute to the development of simple, fast, and efficient methods for analysing eleven varieties of lettuce. However, future studies should focus on incorporating these methods into decision-making systems for precision agriculture, and investigating their potential for monitoring crop quality, detecting diseases and pests, and providing real-time feedback.

## Figures and Tables

**Table 1:** STEPWise models indicating error acceptance and frequency and by model-based VIS-NIR-SWIR comprising reflectance data.

Bands (nm)	Model Test	Frequency (Number)	Percentage (%)
**400–700** **(VIS)**	Error	449	34.5
	Accept	851	65.5
	**Total**	**1300**	**100**
**700–1300 (NIR)**	Error	233	17.8
	Accept	1074	82.2
	**Total**	**1307**	**100**
**1300–2400 (SWIR)**	Error	424	33.1
	Accept	860	66.9
	**Total**	**1284**	**100**
**400–2400** **(VIS-NIR-SWIR)**	Error	282	21.5
	Accept	1030	78.5
	**Total**	**1312**	**100**

## Data Availability

Not applicable.

## Supplementary Materials

The following supporting information can be downloaded at: https://www.mdpi.com/article/10.3390/plants12061333/s1. Table S1. Descriptive analysis parameters of lettuce varieties. Pigment of leaves expressed by leaf area (mg m^−2^), mass (mg g^−1^), and volume (mL L^−1^) (*n* = 132); Table S2. STEPW and VIPs by wavelengths selected according to classified algorithm-based ANOVA and information gain ratio (*p* < 0.001) by band range spectroscopy from reflectance leaves. Bold underlines show shared wavelengths between the STEPW and VIP; Table S3. Description of artificial intelligence algorithms (AIAs).
