# Supplementary material for: Enhancing Pigment Phenotyping and Classification in Lettuce through the Integration of Reflectance Spectroscopy and AI Algorithms
Source: Plants (Basel). 2023 Mar 16;12(6):1333. doi: 10.3390/plants12061333 (PMC10059284; doi:10.3390/plants12061333)
Supplement: Supplementary file 1 [file plants-12-01333-s001.zip › plants-2236572-supplementary.pdf]

plants

Supporting Information

Enhancing Pigment Phenotyping and Classification in Lettuce Through the Integration of Reflectance Spectroscopy and AI Algorithms

Renan Falcioni [1,*], João Vitor Ferreira Gonçalves [1], Karym Mayara de Oliveira [1], Caio Almeida de Oliveira [1], José A. M. Demattê [2], Werner Camargos Antunes [1] and Marcos Rafael Nanni [1]

[1] Graduate Program in Agronomy, Department of Agronomy, State University of Maringá, Av. Colombo, 5790, Maringá 87020-900, Paraná, Brazil
[2] Department of Soil Science, Luiz de Queiroz College of Agriculture, University of São Paulo, Av. Pádua Dias, 11, Piracicaba 13418-260, São Paulo, Brazil
* Correspondence: renanfalcioni@gmail.com; Tel.: +55-44-3011-8940

Table S1. Descriptive analysis parameters of lettuce varieties. Pigment of leaves expressed by leaf area (mg m^{-2}), mass (mg g^{-1}), and volume (mL L^{-1}). (*n* = 132).

Parameters	Count (n)	Mean	Median	Minimum	Maximum	CV (%)
Chl*a* (mg m^{-2})	132	128.3	118.3	63.1	306.4	39.2
Chl*b* (mg m^{-2})	132	66.5	61.7	39.4	147.3	35.1
Chl*a*+*b* (mg m^{-2})	132	194.9	182.1	108.0	453.7	37.6
Car (mg m^{-2})	132	45.5	42.3	25.6	101.9	35.5
AnC (nmol cm^{-2})	132	13.1	11.4	0.0	68.6	125.8
Flv (nmol cm^{-2})	132	58.4	49.0	0.0	178.3	62.2
Chl*a* (mg g^{-1})	132	14.8	14.3	8.2	31.4	26.4
Chl*b* (mg g^{-1})	132	7.7	7.7	4.5	15.1	23.1
Chla+b (mg g^{-1})	132	22.5	21.7	13.2	46.5	24.9
Car (mg g^{-1})	132	5.3	5.1	3.2	10.9	24.2
AnC (µmol g^{-1})	132	16.3	16.4	0.0	83.1	121.3
Flv (µmol g^{-1})	132	74.2	71.0	12.7	203.8	60.1
Phe (mL L^{-1})	132	62.8	66.3	42.7	78.7	16.6
Chl*a*/*b* ratio	132	1.9	2.0	1.4	2.2	9.4
Car/Chl*a*+*b* ratio	132	0.2	0.2	0.2	0.3	6.1

Table S2. STEPW and VIPs by wavelengths selected according to classified algorithm-based ANOVA and information gain ratio (p <0.001) by band range spectroscopy from reflectance leaves. Bold underlines show shared wavelengths between the STEPW and VIP.

Band range (nm)	N	Shared	STEPWise selection wavelengths (nm)	Most responsive VIP-rank selection wavelengths (nm)
**400-700**	34	7	**548**, **570**, **560**, 581, 531, **700**, **544**, 540, 538, 615, **545**, 456, **491**, 437, 434, 645, 654, 452, 429, 472, 641, 609, 687, 658, 687, 658, 614, 626, 430, 594, 441, 528, 527, 659, 427, 443	549, **548**, 550, 547, 551, 546, 552, **545**, **544**, 553, 543, 542, 554, 555, 559, 535, **560**, 533, 573, 522, 524, 574, **570**, 589, 599, 606, 608, 500, 497, 510, **700**, 610, 693, **491**
**700-1300**	57	3	701, 737, 1034, 915, 703, 1274, 933, 975, 995, 924, 922, **954**, 983, 970, 799, 923, 908 1021, 1027, 992, 911, 885, 798, 1088, 1001, 811, 842, 857, 860, 805, 831, 829, 1033, 935, 832, 845, **879**, 928, 801, 780, 787, 807, **986**, 847, 994, 806, 907, 846, 995, 810, 1003, 803, 859, 941, 918, 978, 874	**986**, 965, 960, **954**, 958, 700, 711, 754, 757, 707, 766, 749, 762, 748, 767, 1057, 1156, 1295, 1151, 1149, 1445, 1169, 1173, 1284, 789, 1283, 1174, 1131, 1276, 795, 1185, 797, 1115, 1266, 1108, 896, 814, 913, 1423, 1055, 1299, 1047, 861, 1058, 1249, 1109, 743, 867, 1087, 1244, **879**, 745, 892, 740, 715, 768, 853
**1300-2400**	22	2	2245, 1861, 2023, 1310, 1830, 1842, 2220, 1921, 1803, 1806, 1841, **1816**, 1941, 2277, 2295, 2296, 1829, 1843, **2013**, 1924, 1837, 1810	2364, 2371, 2366, 2373, 2400, 2353, 1442, 1437, **1816**, 2350, 1836, 1842, 1460, 1887, **2013**, 1385, 2324, 2088, 1416, 1789, 1658, 1781
**400-2400**	26	3	**548**, 570, **560**, 581, 531, 701, 544, 540, 743, 1023, 952, 527, 1059, 741, **1539**, 1754, 700, 942, 1009, 998, 1065, 562, 559, 1000, 574, 597	**548**, 549, 550, 547, 551, 546, **560**, 553, 572, 583, 2378, 2373, 2367, 2382, 2326, 600, 2045, 2071, 1435, 1425, 1993, 1446, 1840, 1940, **1539**, 435

Table S3. Description of artificial intelligence algorithms (AIAs).

Algorithms	Abbreviation	Description	References

AdaBoost	AdB	Boosting algorithm that combines weak learners to create a strong classifier.	[41]
CN2 rule inducer	CN2	Decision tree algorithm that creates a set of if-then rules to classify data.	[41]
Constant	Const	Algorithm that always predicts the same class, regardless of input.	[41]
Gradient Boosting	G-Boo	Boosting algorithm that combines multiple decision trees to create a strong classifier.	[41]
Kernel k Nearest Neighbours	KNN	Non-parametric algorithm that classifies data based on the k closest training examples in a feature space.	[41]
Logistic Regression	Log-Reg	Parametric algorithm that models the probability of a binary outcome based on input features.	[41]
Naive Bayes	Nai-Bay	Probabilistic algorithm that models the likelihood of each class given the input features.	[41]
Neural Network	NN	Algorithm that models complex non-linear relationships between inputs and outputs using layers of interconnected nodes.	[41]
Stochastic Gradient Descent	SGD	Optimization algorithm that minimizes the error of a model by adjusting the weights of the inputs.	[41]
Random Forest	RF	Ensemble learning algorithm that combines multiple decision trees to create a strong classifier.	[41]
Support Vector Machines	SVM	Algorithm that finds the hyperplane that best separates two classes in a feature space.	[41]
Tree	Tree	Decision tree algorithm that recursively partitions data based on input features.	[41]

*** references were based explication of models.

41. Https://orangedatamining.com/widget-catalog/ Orange Models.
